# Isocitrate Dehydrogenase-1 Mutations as Prognostic Biomarker in Glioblastoma Multiforme Patients in West Bohemia

**DOI:** 10.1155/2014/735659

**Published:** 2014-01-09

**Authors:** J. Polivka, J. Polivka, V. Rohan, M. Pesta, T. Repik, P. Pitule, O. Topolcan

**Affiliations:** ^1^Department of Neurology, Faculty of Medicine in Pilsen, Charles University in Prague, Husova 3, 301 66 Pilsen, Czech Republic; ^2^Faculty Hospital in Pilsen, Alej Svobody 80, 304 60 Pilsen, Czech Republic; ^3^Department of Histology and Embryology and Biomedical Centre, Faculty of Medicine in Pilsen, Charles University in Prague, Husova 3, 301 66 Pilsen, Czech Republic; ^4^Department of Biology, Faculty of Medicine in Pilsen, Charles University in Prague, Husova 3, 301 66 Pilsen, Czech Republic; ^5^Central Immunoanalytical Laboratory, Faculty Hospital in Pilsen, Dr. E. Benese 13, 305 99 Pilsen, Czech Republic

## Abstract

*Introduction*. Glioblastoma multiforme (GBM) is the most malignant primary brain tumor in adults. Recent whole-genome studies revealed novel GBM prognostic biomarkers such as mutations in metabolic enzyme IDH—isocitrate dehydrogenases (IDH1 and IDH2). The distinctive mutation IDH1 R132H was uncovered to be a strong prognostic biomarker for glioma patients. We investigated the prognostic role of IDH1 R132H mutation in GBM patients in West Bohemia. *Methods*. The IDH1 R132H mutation was assessed by the RT-PCR in the tumor samples from 45 GBM patients treated in the Faculty Hospital in Pilsen and was correlated with the progression free and overall survival. *Results*. The IDH1 R132H mutation was identified in 20 from 44 GBM tumor samples (45.4%). The majority of mutated tumors were secondary GBMs (16 in 18, 89.9%). Low frequency of IDH1 mutations was observed in primary GBMs (4 in 26, 15.3%). Patients with IDH R132H mutation had longer PFS, 136 versus 51 days (*P* < 0.021, Wilcoxon), and OS, 270 versus 130 days (*P* < 0.024, Wilcoxon test). *Summary*. The prognostic value of IDH1 R132H mutation in GBM patients was verified. Patients with mutation had significantly longer PFS and OS than patients with wild-type IDH1 and suffered more likely from secondary GBMs.

## 1. Introduction

Glioblastoma multiforme (GBM) is the most common and most malignant primary brain tumor in adults with an incidence of 3-4/100,000/year. The median survival of patients with GBM is 12.1–14.6 months [1] and only 3–5% of patients survive longer than 3 years [2, 3]. The progress in genomics of GBM has revealed several abnormalities in signaling pathways and a diversity of mutated genes. One of great importance among them is isocitrate dehydrogenase (IDH) [4, 5]. Isocitrate dehydrogenases (three isoforms IDH1, IDH2, and IDH3) catalyze the oxidative carboxylation of isocitrate to alpha-ketoglutarate and reduce nicotinamide adenine dinucleotide phosphate (NADP) to NADPH, which is necessary for the regeneration of reduced glutathione that serves as the main antioxidant [6]. The genes for IDH1 and IDH2 carry specific mutations in 70%–80% of low-grade gliomas, in approximately 50% of anaplastic gliomas, and in more than 5% of glioblastomas [7, 8]. The mutations are involved in 90% single amino acid substitution—R132H in the IDH1 active site that leads to the loss of regular enzyme function—and are predominantly heterozygous. Mutations in IDH2 occurred rarely in brain tumors [7, 9]. The aberrant function of mutated IDH1 is the conversion of alpha-ketoglutarate to the novel oncometabolite 2-hydroxyglutarate (2-HG), which leads to genome-wide epigenetic changes in human gliomas [10]. Tumors with mutated IDH1 and corresponding epigenetic changes demonstrated better prognosis than gliomas with wild-type IDH1. This association was observed also for GBM [4, 6, 7, 11, 12]. The aim of this study was to assess the prognostic role of IDH1 R132H mutation in the relation to progression-free survival (PFS) as well as overall survival (OS) of our GBM patients in West Bohemia.

## 2. Patients and Methods

### 2.1. Patients

We performed a retrospective study of 44 patients with a diagnosis of WHO grade IV astrocytoma—GBM (*n* = 44; 22 males and 22 females; mean age 64.3 years) who were treated (total or subtotal tumor resection or tumor biopsy, radiotherapy, and chemotherapy with temozolomide) in the Faculty Hospital in Pilsen between the years 2009 and 2011. The study protocol was approved by the ethics committee (Table 1).

### 2.2. DNA Isolation

DNA was extracted from 10 *μ*m FFPE sections following macrodissection of tumor tissue and normal brain tissue using the QIAamp DNA FFPE Tissue kit (Qiagen, Hilden, Germany). The 10 *μ*m sections corresponded to HES-representative with tumor tissue verified by pathologist.

### 2.3. Mutation Detection

For detection of mutant allele IDH1 c.395G>A (p.R132H, COSMIC ID 28746), we use TaqMan Mutation Detection Assays (assay name: IDH1 28746 mu and IDH1 rf) with the TaqMan Mutation Detection IPC Reagent Kit (Life Technologies, Carlsbad, California, USA). Mutant allele detection we performed according to recommended procedure and reaction conditions is found in the manual. For the amplification, we used the Stratagene Mx3000P real-time PCR system instrument (Agilent Technologies, Inc., Santa Clara, CA, USA). Detection of mutant alleles was performed in duplicate in a reaction volume of 20 *µ*L. Detection of reference gene was also performed in duplicate. The analysis of the positive samples was repeated. Before analysis of our collection of tumor samples, we analyzed samples of normal brain tissue for detection of cut-off amplification curve. No amplification of mutant allele was present in normal brain tissue. On the basis of these results and the shape of amplification curve of positive tumor samples, we determined the ΔCt cut-off 25 value.

### 2.4. Statistical Analysis

Overall survival (OS) was defined as the time between the diagnosis and death or last follow-up. Progression-free survival (PFS) was defined as the time between the diagnosis and recurrence or last follow-up. Kaplan-Meier survival curves were plotted and the survival distributions were compared with the use of the Wilcoxon test. Reported *P* values are two-sided. *P* values of less than 0.05 were considered to indicate statistical significance.

## 3. Results

**Figure 1:**
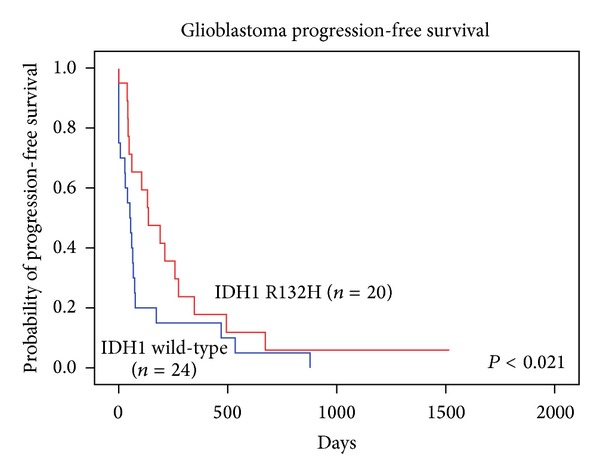
Progression-free survival of patients with glioblastoma with (red line) or without (blue line) IDH1 R132H gene mutation.

**Figure 2:**
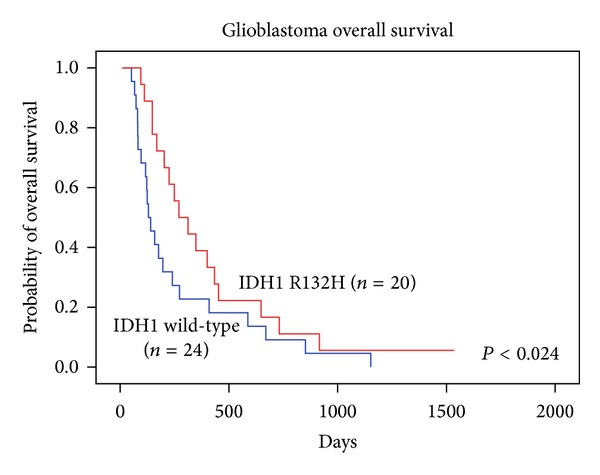
Overall survival of patients with glioblastoma with (red line) or without (blue line) IDH1 R132H gene mutation.

The examined mutation IDH1 R132H was observed in 20 of 44 GBM-patient tumor samples. Therefore we identified the IDH1 mutation in more than 45.4% of glioblastomas. The separation of primary and secondary glioblastomas (GBM that progressed from the low-grade glioma) was done on the basis of clinically relevant information, where possible. The IDH1 R132H mutation occurred in 4 of 26 primary GBMs (15.3%), whereas the majority, 16 of 18 (89.9%) were of secondary glioblastomas mutated (Table 2). The significant relation between IDH1 mutation status and clinical parameters such as PFS and OS was also observed (Table 3). Patients with IDH1 R132H mutation had longer PFS than patients with wild-type IDH1-136 versus 51 days (*P* < 0.021, Wilcoxon test) (Figure 1). Significantly longer OS was observed as well for patients with IDH1 R132H mutation than for patients without the mutation-270 versus 130 days (*P* < 0.024, Wilcoxon test) (Figure 2).

## 4. Discussion

**Figure 3:**
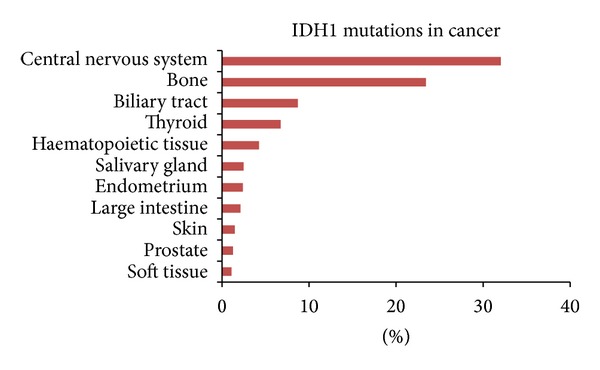
The representation of IDH1 mutations in various cancers [14].

**Figure 4:**
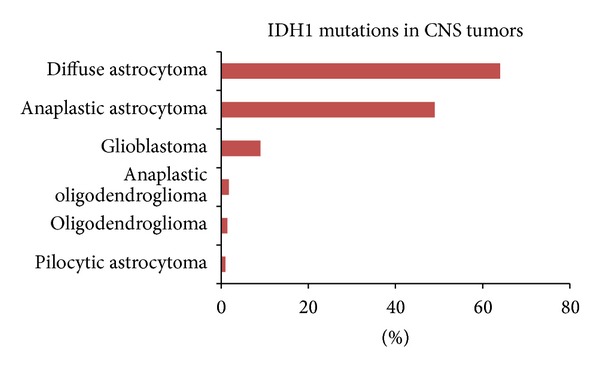
The representation of IDH1 mutations in various types of central nervous system tumors [14].

Recurrent IDH mutations and their role in oncogenesis and tumor progression were described for the first time in GBM [4]. This observation has led to new insights into GBM and cancer biology. Alterations in cancer cell metabolism are now well accepted as one of the principal hallmarks of the cancerogenesis and tumor progression [13]. Mutations in IDH1 were also identified in substantial portion of other tumor types. The data from the Sanger Institute Cancer Genome Project-Catalogue of Somatic Mutations in Cancer revealed the presence of IDH1 mutations in more than 32% of central nervous system tumors, 23% of bone tumors, 8% of biliary tract tumors, 6% of thyroid cancer, and many other tumor types [14] (Figure 3). In the primary brain tumors group, IDH1 mutations are presented mostly in diffuse astrocytomas (64%), anaplastic astrocytomas (49%), glioblastomas (9%), or oligodendrogliomas (2%) [14] (Figure 4). The R132H amino acid substitution is the most common form of IDH1 mutations with the prevalence of 90% among IDH1-mutant tumors. Less common mutants such as R132C, R132G, R132S, and R132L are also known [7, 9].

The fundamental shift in the understanding of mutated IDH and its role in cancer progression came with the observation of the neomorphic function of the mutated enzyme. Instead of the production of alpha-ketoglutarate, mutated IDH1 produced novel oncometabolite 2-hydroxyglutarate (2-HG) that was highly accumulated in the cancer cells [15]. It was subsequently discovered that 2-HG inhibits the functions of the alpha-ketoglutarate dependent superfamily of dioxygenases. These enzymes have diverse cellular functions including, but not limited to, histone demethylation and demethylation of hypermethylated DNA [16, 17]. Moreover, IDH1 mutations and 2-HG production were identified to be sufficient steps in the process leading to glioma hypermethylator phenotype. That observation was important for understanding of glioma oncogenesis and highlighted the interplay between genomic and epigenomic changes in human cancers [10, 18].

Mutations in IDH1 are important also for their clinical consequences. Recent studies revealed the important role of mutated IDH1 in the assessment of astrocytoma patient prognosis. Therefore IDH1 mutations could serve in the near future as the standard prognostic biomarkers for patients with grade II, III, and IV astrocytomas. The differences in OS between IDH1-mutant and IDH1 wildtype GBM were 3.8 versus 1.1 years [4], 2.6 versus 1.3 years [7], 2.3 versus 1.2 years [6], and 3 years versus 1 year in several studies [11]. Similar OS differences in IDH1-mutant versus IDH1-WT tumors were observed for anaplastic astrocytomas, such as 5.4 versus 1.7 years [7], 6.8 versus 1.6 years [6], and 7 versus 2 years [11] as well as for low-grade gliomas [19]. Recent meta-analysis also confirmed the prognostic role of IDH1/2 mutations in gliomas [20]. These data highlight the major impact of IDH1 mutation status on glioma patient survival and support the incorporation of this biomarker into the clinical assessments. Mutations in IDH1/IDH2 and production of oncometabolite 2-HG could be used as well for therapeutic intervention in the near future [21].

The results from our study also support the IDH1 mutation R132H to be the strong prognostic factor for patients with GBM. Although the differences in median PFS and OS between patients with IDH1 mutated and IDH1 wild-type tumors are not as big as in other studies, they are statistically significant. One reason for the relatively small differences in median PFS and OS between both groups could be the heterogeneity of the treatment protocols. The standard treatment with neurosurgery and concomitant chemo-radiotherapy with temozolomide was implemented in 29 patients and 1 patient had only radiotherapy and 15 patients were treated neither with radiotherapy nor with chemotherapy (Table 1). The proportion of IDH1 mutated tumors is also higher in our study than in other similar studies. The IDH1 mutations in glioblastomas were formerly identified predominantly in secondary GBM that progressed from the low grade tumors [22]. In our study, we tried to distinguish between the primary and secondary glioblastomas on the basis of clinically relevant information from the patient history. However, the distinction between primary and secondary GBM was not possible exactly. Only 5 patients had previously assessed low grade glioma (surgery in 2 cases, tumor biopsy in 3 cases). Patients with tumor corresponding neurological symptomatology (epileptic seizures, focal neurological deficit) present at least 6 months before the tumor diagnosis was considered as likely secondary GBM. Moreover the primary-like glioblastomas could be in fact secondary without the symptoms of low grade tumors.

The recent study of mutations in telomerase reverse transcriptase (TERT) gene promoter has revealed the high incidence of these aberrations in a large portion of primary GBMs (about 80%) [23]. In the perspectives of our further research, we will use TERT promoter mutations in addition to clinically relevant information for the separation of primary and secondary glioblastomas. The assessment of other IDH1 mutations than R132H as well as the analysis of mutations in IDH2 is also planned.

Despite the drawbacks of our study mentioned above, IDH1 R132H mutation still serves as a strong prognostic biomarker for our patients with GBM.

## 5. Summary

The IDH1 R132H mutation was observed in the interestingly higher number of patients with GBM that was previously published by other groups. On the other hand, the majority of mutated GBMs in our cohort are probably secondary glioblastomas. The prognostic value of the IDH1 R132H mutation was also observed. Patients with this mutation had significantly longer PFS as well as OS than patients with wild-type IDH1. The IDH1 mutation status could be used as a strong prognostic factor for patients with GBM, but further validation of this biomarker in large prospective clinical trials is urgently needed.

## Figures and Tables

**Table 1:** Glioblastoma patient demographics and clinical characteristics.

Patient characteristics	
Sex	
Male to female	1
Male	22
Female	22
Age, years	
Median	64.3
Range	35–87
KPS	
Median	77.5
Range	30–100
Postoperative treatment	
RT (±CT)	29
CT alone	1
None	15

KPS: Karnofsky performance score; RT: radiotherapy; CT: chemotherapy.

**Table 2:** The representation of IDH1 R132H mutation in primary versus secondary glioblastomas.

Glioblastoma type	Primary GBM (*n* = 26)	Secondary GBM (*n* = 18)
Mutation status [*n*]		
IDH1 R132H	4 (15.3%)	16 (89.9%)
IDH1 wild-type	22 (84.7%)	2 (11.1%)

**Table 3:** Results for progression-free survival and overall survival differences in patients with GBM in relation to IDH1 mutation status.

Glioblastoma results	*N*	Median [days] (95% Cl)	*P* (Wilcoxon)
Overall survival (OS)			
IDH1 R132H	20	270 (139–400)	0.024
IDH1 wild-type	24	130 (87–172)	
Progression-free survival (PFS)			
IDH1 R132H	20	136 (22–249)	0.021
IDH1 wild-type	24	51 (19–82)	
